# Metabolic Reprogramming in SARS-CoV-2 Infection Impacts the Outcome of COVID-19 Patients

**DOI:** 10.3389/fimmu.2022.936106

**Published:** 2022-07-11

**Authors:** Laura E. Martínez-Gómez, Isabel Ibarra-González, Cynthia Fernández-Lainez, Teresa Tusie, Hortensia Moreno-Macías, Carlos Martinez-Armenta, Guadalupe Elizabeth Jimenez-Gutierrez, Paola Vázquez-Cárdenas, Patricia Vidal-Vázquez, Juan P. Ramírez-Hinojosa, Ana P. Rodríguez-Zulueta, Gilberto Vargas-Alarcón, Gustavo Rojas-Velasco, Fausto Sánchez-Muñoz, Rosalinda Posadas-Sanchez, Felipe de J. Martínez-Ruiz, Dulce M. Zayago-Angeles, Mariana L. Moreno, Edith Barajas-Galicia, Gerardo Lopez-Cisneros, Nadia C. Gonzalez-Fernández, Silvestre Ortega-Peña, Brígida Herrera-López, Jessel Olea-Torres, Manuel Juárez-Arias, Maritza Rosas-Vásquez, Sara Aileen Cabrera-Nieto, Jonathan J. Magaña, María del Carmen Camacho-Rea, Carlos Suarez-Ahedo, Irma Coronado-Zarco, M. Y. Valdespino-Vázquez, Gabriela Angélica Martínez-Nava, Carlos Pineda, Marcela Vela-Amieva, Alberto López-Reyes, Mex-Gen-COVID Initiative Group

**Affiliations:** ^1^Laboratorio de Gerociencias, Laboratorio Facilitador, Laboratorio de Medicina Genómica, Dirección General, Instituto Nacional de Rehabilitación Luis Guillermo Ibarra Ibarra, Secretaría de Salud, Ciudad de México, Mexico; ^2^Unidad de Genética de la Nutrición, Instituto de Investigaciones Biomédicas, Universidad Nacional Autónoma de México (UNAM), Ciudad de México, Mexico; ^3^Laboratorio de Errores Innatos del Metabolismo y Tamiz, Instituto Nacional de Pediatría, Secretaría de Salud, Ciudad de México, Mexico; ^4^Unidad de Biología Molecular y Medicina Genómica, Instituto Nacional de Ciencias Médicas y Nutrición Salvador Zubirán, Instituto de Investigaciones Biomédicas UNAM, Ciudad de México, Mexico; ^5^Departamento de Economía. División de Ciencias Sociales y Humanidades, Universidad Autónoma Metropolitana, Iztapalapa, Ciudad de México, Mexico; ^6^Posgrado en Biología Experimental, Dirección de Ciencias Biológicas y de la Salud (DCBS), Universidad Autónoma Metropolitana Iztapalapa, Ciudad de México, Mexico; ^7^Centro de Innovación Médica Aplicada, Subdirección de Epidemiología e Infectología, Hospital General Dr. Manuel Gea González, Secretaría de Salud, Ciudad de México, Mexico; ^8^Departamentos de Biología Molecular, Inmunología, Endocrinologia y Unidad de Cuidados Intensivos, Instituto Nacional de Cardiología Ignacio Chavez, Secretaría de Salud, Ciudad de México, Mexico; ^9^Nuevo Hospital General Delegación Regional Sur de la Ciudad de México, Instituto de Seguridad y Servicios Sociales para los Trabajadores del Estado (ISSSTE), Ciudad de México, Mexico; ^10^Hospital Central Norte Petróleos Mexicanos (PEMEX), Estado de México, Mexico City, Mexico; ^11^Unidad de Investigación y Desarrollo en Alimentos, Tecnológico Nacional de México/Instituto Tecnológico (IT) Veracruz, Veracruz, Mexico; ^12^Posgrado en Ciencias Médicas, Facultad de Ciencias de la Salud, Universidad Anáhuac, Ciudad de México, Mexico; ^13^Departamento de Nutrición Animal, Instituto Nacional de Ciencias Médicas y Nutrición Salvador Zubirán, Secretaría de Salud, Ciudad de México, Mexico; ^14^Instituto Nacional de Perinatología Isidro Espinosa de los Reyes, Ciudad de México, Mexico

**Keywords:** COVID-19, metabolomics, SARS – CoV – 2, amino acids, phenylalanine

## Abstract

Severe acute respiratory syndrome–coronavirus 2 (SARS-CoV-2) infection triggers inflammatory clinical stages that affect the outcome of patients with coronavirus disease 2019 (COVID-19). Disease severity may be associated with a metabolic imbalance related to amino acids, lipids, and energy-generating pathways. The aim of this study was to characterize the profile of amino acids and acylcarnitines in COVID-19 patients. A multicenter, cross-sectional study was carried out. A total of 453 individuals were classified by disease severity. Levels of 11 amino acids, 31 acylcarnitines, and succinylacetone in serum samples were analyzed by electrospray ionization–triple quadrupole tandem mass spectrometry. Different clusters were observed in partial least squares discriminant analysis, with phenylalanine, alanine, citrulline, proline, and succinylacetone providing the major contribution to the variability in each cluster (variable importance in the projection >1.5). In logistic models adjusted by age, sex, type 2 diabetes mellitus, hypertension, and nutritional status, phenylalanine was associated with critical outcomes (odds ratio=5.3 (95% CI 3.16-9.2) in the severe vs. critical model, with an area under the curve of 0.84 (95% CI 0.77-0.90). In conclusion the metabolic imbalance in COVID-19 patients might affect disease progression. This work shows an association of phenylalanine with critical outcomes in COVID-19 patients, highlighting phenylalanine as a potential metabolic biomarker of disease severity.

## Introduction

The severe acute respiratory syndrome–coronavirus 2 (SARS-CoV-2) pandemic has drastically impacted humanity, threatening public health and the global economy (1). Infections of pneumocytes by SARS-CoV-2 is responsible for causing coronavirus disease 2019 (COVID-19) (2), which has infected approximately 458 million people worldwide and caused the death of more than 6 million people (3). Reported clinical manifestations of COVID-19 range from asymptomatic infection to mild, severe, and critical disease (4). Patients with critical outcomes develop dyspnea, hypoxia, acute respiratory disease syndrome, multiple organ dysfunction syndrome (5), septic and cardiogenic shock, and acute myocardial injury or myocarditis (6–8). Cytokine storm, iron overload, anemia, and hypoxia have been proposed as possible factors related to critical outcomes and death (9–14).

Various aspects of the host-virus interaction have been proposed as part of the mechanism to explain the complexity of COVID-19 pathogenesis (15). In this sense, the virus induces modifications in host metabolism, including pathways related to amino acids, lipids, and energy generation, leading to metabolic reprogramming that can be reflected as an impaired metabolome (16–18). Recent evidence indicates that SARS-CoV-2 causes metabolic dysregulation at different levels, affecting glucose, cholesterol, amino acid, and fatty acid metabolism (17–19). Some of these observed metabolic changes in the host could be related to an increase in muscular protein and lipid catabolism as a means of meeting the high energy requirement needed to fight the infection (20). Recent studies have shown that phenylalanine metabolism is one of the most dysregulated pathways in COVID-19 patients (17, 20–22). However, the effects of SARS-CoV-2 infection on intermediary metabolism, including metabolism of acylcarnitines, has been poorly studied. Nevertheless, measurement of total carnitine has been used as a precision biomarker to predict mortality risk in diseases such as sepsis, T2DM, cancer, and heart failure (23).

Another key metabolite that has been poorly explored in COVID-19 is succinylacetone. This metabolite is an organic acid which comes from tyrosine catabolism and when fumarylacetoacetate hydrolase enzyme activity is impaired it can accumulate contributing to acidosis, it can also act as an oncometabolite, or a metabotoxin (24). A better understanding of dysregulation of amino acid, succinylacetone and acylcarnitine metabolism associated with the different COVID-19 clinical phenotypes could provide novel insights into treatment strategies that reduce the index of fatal outcomes. The present study aimed to characterize the profile of amino acids, succinylacetone, and acylcarnitines in a cohort of COVID-19 patients as potential biomarkers of different stages of the disease.

## Material and Methods

### Setting and Participants

From June 2020 to March 2021, a cross-sectional multicenter study was carried out. The inclusion criteria for selecting subjects were independent of gender, age ≥18 years, non-vaccinated, and non-pregnant women with clinical manifestations of COVID-19 and positive RT-PCR test. A non-probabilistic sampling study design was used, as patients were recruited directly from the COVID-19 triage facilities of the participant institutions. The participants were recruited from the following public hospitals of the Mexican Governmental Health System in Mexico City: Instituto Nacional de Rehabilitación “Luis Guillermo Ibarra Ibarra”, Instituto Nacional de Cardiología “Ignacio Chávez”, Hospital Central Norte Pemex, Instituto Nacional de Ciencias Médicas y Nutrición “Salvador Zubirán”, Hospital General “Dr. Manuel Gea González”, and Hospital General ISSSTE “Tláhuac”.

### Ethical Statement

This study was conducted following good clinical practices and the Declaration of Helsinki. Written informed consent was obtained from each participant or legal representative. The privacy of patient data was stated at the time of informed consent. The ethics and research committees of the participating institutions approved this study.

### Outcomes

Patients were classified by disease severity according to Gandhi et al. criteria (4), as follows: mild (n=152), ambulatory subjects with symptoms such as fever, headache, fatigue, odynophagia, cough, rhinorrhea, diarrhea, anosmia, or dysgeusia, with or without dyspnea or pneumonia, not requiring hospitalization; severe (n=60), hospitalized individuals with any of the following symptoms: tachypnea (respiratory rate>30 bpm); pulmonary infiltrate >50%, dyspnea after small efforts; and critical (n=210), patients requiring invasive mechanical ventilation who could course to shock and multi-organ failure. In addition, the healthy group was selected by the surgery orthopedic service at the Instituto Nacional de Rehabilitación LGII, including subjects with negative RT-PCR test to SARS-CoV-2 and without symptoms of fever, odynophagia, myalgia, anosmia. Thus, four groups were studied: healthy, mild, severe, and critical patients.

### Sample and Metabolomic Analysis

Venous blood samples were obtained mainly at hospital COVID-19 triage, or in the first hours of admission. Samples were immediately centrifugated and serum was separated and stored at -70 oC. Targeted metabolomic analysis was performed using a commercial kit (NeoBaseTM non-derivatized MSMS kit, Perkin Elmer-WallacTM Oy, Turku, Finland), which comprises eleven amino acids (alanine, arginine, citrulline, glycine, XLE-OHPro [leucine, isoleucine alloisoleucine and hydroxyproline], methionine, ornithine, phenylalanine, proline, tyrosine, and valine); 31 acylcarnitines (Free-carnitine, acetylcarnitine, propionylcarnitine, malonylcarnitine, butyrylcarnitine, 3-hydroxy-butyrylcarnitine, methylmalonylcarnitine, isovalerylcarnitine, tiglylcarnitine, glutarylcarnitine, 3-hydroxy-isovalerylcarnitine, hexanoylcarnitine, adipylcarnitine, octanoylcarnitine, octenoylcarnitine, decanoylcarnitine, decenoylcarnitine, dodecanoylcarnitine, dodecenoylcarnitine, tetradecanoylcarnitine, tetradecenoylcarnitine, tetradecadienoylcarnitine, 3-hydroxy-tetradecanoylcarnitine, hexadecanoylcarnitine, hexadecenoylcarnitine, 3-hydroxy-hexadecanoylcarnitine, 3-hydroxy-hexadecenoylcarnitine, octadecanoylcarnitine, octadecenoylcarnitine, octadecadienoylcarnitine, 3-hydroxy-octadecanoylcarnitine, 3-hydroxy-octadecenoylcarnitine, and one organic acid (succinylacetone). All the reagents for the measurement such as internal and calibration standards, quality controls and the mobile phase were included in the kit. Samples were processed following the manufacturer’s instructions. Briefly, metabolites were extracted from samples using a methanolic solution that included stable isotope–labeled standards for quantification. After extraction, the samples were analyzed with a MSMS instrument (MSMS, Quattro micro-API, Waters Inc, Milford, MA, USA), which is constituted of a triple quadrupole mass analyzer. The samples were introduced without previous chromatographic step, by flow injection. Analytes were ionized by electrospray ionization using nitrogen as curtain gas. The collision gas used between the two mass spectrometers was argon. For data acquisition multiple reaction monitory method was used. Metabolites were quantified with NeoLynxTM software (Perkin Elmer-WallacTM Oy, Turku, Finland).

MetaboAnalyst version 5.0 was used. Data were normalized according to the constant sum method, and data scaling was performed with mean-centering and division by the standard deviation of each variable. Data transformation was performed using the log2-normalized median fold-change and the level of each amino acid was calculated and compared with the level in healthy subjects (HS). Hierarchical clustering was performed using the so-called h-clust function in the package stat, which is included in the MetaboAnalyst software. Clustering results are shown as a heatmap. Partial least squares discriminant analysis (PLS-DA) was used to identify metabolites that could outline different clusters related to disease severity among all the studied groups. The variable importance in projection (VIP) based on de PLS-DA analysis was determined to identify metabolites that would enable discrimination of disease severity, those metabolites with VIP score ≥1.5 were considered significant for group separation (25, 26).

### Statistical Analysis

Clinical and anthropometric analyses: normality of the variables was determined using the Kolmogorov-Smirnov test. Univariate and bivariate exploratory analyses were also carried out. The Kruskal-Wallis test was used for nonparametric continuous variables, and results are described using the median and interquartile range (IQR). For categorical variables, chi-squared or Fisher exact tests were performed. A value of p<0.05 was considered statistically significant for all tests.

To associate the COVID-19 phenotypes to those metabolites with a VIP>1.5, we performed unadjusted and adjusted logistic regression models. For this analysis the reference group used was the mild disease group. Age, sex, type 2 diabetes mellitus (T2DM), hypertension, and body mass index (BMI) according to WHO criteria (≥18.5-24.9: normal weight; ≥25-29.9: overweight and ≥30: obesity) were considered as covariates. Receiver-operating characteristic (ROC) curves were used to determine sensitivity, specificity, and area under the curve (AUC). Bonferroni correction for multiple comparisons was performed, and p ≤ 0.003 was considered statistically significant. All analyses were carried out using the STATA 13 statistical package.

## Results

### Subjects

This cross-sectional, multicenter study included 453 non-vaccinated subjects, 31 of whom were healthy subjects (HS) that formed the control group, and 422 who formed the COVID-19 group and presented clinical characteristics of COVID-19, which was confirmed by RT-PCR diagnosis of SARS-CoV-2 infection. Males represented 59% (n=267) of the study population. The median age of COVID-19 patients was 51 years (IQR=41-62 years). We found significant differences among the study groups in terms of anthropometric and clinical characteristics. For instance, a significant reduction in oxygen saturation with increasing disease severity was observed. Moreover, overweight, obesity, T2DM, and hypertension were more prevalent in severe and critical COVID-19 patients (**Table 1**).

**Table 1:** Anthropometric and clinical characteristics of the studied population.

	Healthy n = 31	Mild n = 152	Severe n = 60	Critical n = 210	P value
Age (years) ^&^	43 (32,52)	42 (31,49)	51.5 (44,63.5)	57(49,67)	<0.001**
Male gender, n (%)	17 (55)	73 (48)	33 (55)	144(69)	0.001*
Heart rate, median (IQR), bpm^+&^	85 (73,102)	87 (78,103)	90 (80,103)	95 (88,110)	<0.001**
Body temperature, median (IQR) ^&^, °C	36.3 (36.3,36.45)	36.35 (36.1,36.5)	37 (36.4,38)	36.5 (36.1,37.1)	<0.001**
Oxygen saturation % (IQR) ^&^	95 (94,97)	94 (93,96)	88 (80,92)	83 (71,88)	<0.001**
Breathing frequency, median (IQR), bpm+&	16 (15,16.5)	18 (16,20)	18 (17,20)	28 (24,36)	<0.001**
WHO body mass index classification, n (%)					
Normal weight	17 (55)	115 (76)	20 (33)	37 (18)	<0.001*
Overweight	11 (35)	21 (14)	22 (37)	88 (42)	
Obesity	3 (10)	16 (10)	18 (30)	85 (40)	
T2DM, n (%)	3 (13)	13 (8)	21 (35)	74 (35)	<0.001*
Hypertension, n (%)	4 (17)	11 (7)	15 (25)	85 (41)	<0.001*
Fever, n (%)	0 (0)	47 (31)	29 (48)	116 (57)	<0.001*
Cough, n (%)	5 (16)	90 (59)	44 (73)	171 (81)	<0.001*
Dyspnea, n (%)	1 (3)	39 (26)	43 (72)	152 (72)	<0.001*
Chest pain, n (%)	1 (3)	37 (24)	19 (36)	60 (30)	0.006*
Headache, n (%)	3 (10)	95 (62)	30 (50)	125 (59)	<0.001*
Odynophagia, n (%)	0 (0)	74 (49)	29 (48)	82 (39)	<0.001*
Rhinorrhea, n (%)	4 (13)	67 (44)	9 (15)	42 (20)	<0.001*
Myalgia, n (%)	0 (0)	78 (67)	24 (57)	113 (54)	<0.001*
Diarrhea, n (%)	2 (6)	37 (24)	17 (28)	53 (25)	0.11*
Sickness, n (%)	1 (3)	33 (22)	3 (5)	104 (49)	<0.001*
Vomiting, n (%)	0 (0)	6 (4)	4 (7)	22 (10)	0.09*
Abdominal pain, n (%)	0 (0)	18 (12)	8 (13)	25 (12)	0.82*
Anosmia, n (%)	0 (0)	33 (22)	13 (22)	24 (11)	0.004*

^+^Beats per minute; *Chi square test; **Kruskal-Wallis Test. IQR, interquartile range.

^&^Dunn post hoc test p<0.05. Age: Healthy vs Severe; Healthy vs. Critical; Mild vs. Sever; Mild vs. Critical; Severe vs. Critical.

^&^Dunn post hoc test p<0.05. Heart rate: Mild vs. Critical.

^&^Dunn post hoc test p<0.05. Body temperature: Healthy vs Severe; Mild vs. Sever; Mild vs. Critical; Severe vs. Critical.

^&^Dunn post hoc test p<0.05. Oxygen saturation: Healthy vs Severe; Healthy vs. Critical; Mild vs. Sever; Mild vs. Critical; Severe vs. Critical.

^&^Dunn post hoc test p<0.05. Breathing frequency: Healthy vs. Critical; Mild vs. Sever; Sever vs. Critical.

### The Metabolomic Characterization

**Figure 1:**
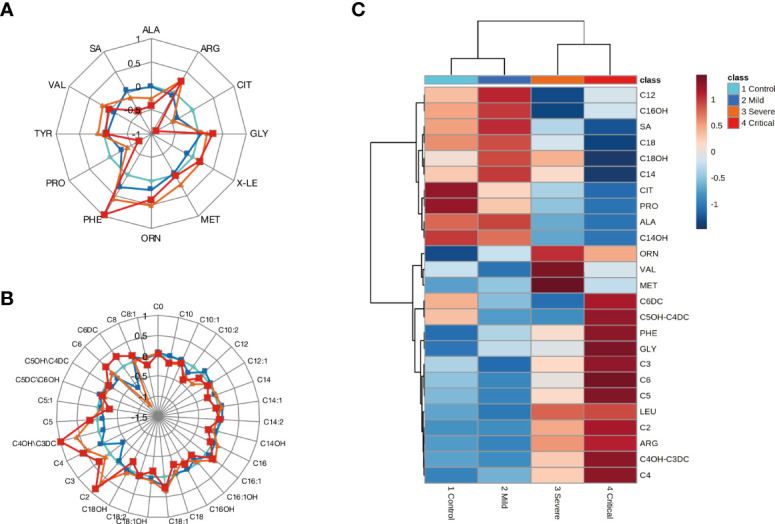
Changes associated with COVID-19 clinical severity. **(A)** Radar plot of amino acid and SA profiles in the studied groups. **(B)** Radar plot of acylcarnitine profiles in the studied groups. The log2-normalized median fold-change in the levels of acylcarnitines was calculated and compared with the levels in HS. **(C)** Hierarchical clustering analysis heatmap illustrating the changes in serum metabolite abundance (average serum metabolites from each studied group) of the top 25 metabolites from HS and patients with mild, moderate, and critical COVID-19. The colored boxes on the right of the figure indicate the relative concentrations of the corresponding metabolite in each group under study, from less concentrated (dark blue) to most concentrated (dark red). ALA, Alanine; ARG, Arginine; CIT, Citrulline; GLY, Glycine; XLE-OHPro (Leucine, Isoleucine Alloisoleucine and Hydroxyproline); MET, Methionine; ORN, Ornithine; PHE, Phenylalanine; PRO, Proline; TYR, Tyrosine; VAL, Valine; SA, Succinylacetone; C0, Free-carnitine; C2, Acetylcarnitine; C3, Propionylcarnitine; C3DC, Malonylcarnitine; C4, Butyrylcarnitine; C4OH, 3-hydroxy-butyrylcarnitine; C4DC, Methylmalonylcarnitine; C5, Isovalerylcarnitine; C5:1, Tiglylcarnitine; C5DC, Glutarylcarnitine; C5OH, 3-Hydroxy-isovalerylcarnitine; C6, Hexanoylcarnitine; C6DC, Adipylcarnitine; C8, Octanoylcarnitine; C8:1, Octenoylcarnitine; C10, Decanoylcarnitine; C10:1, Decenoylcarnitine; C12, Dodecanoylcarnitine; C12:1, Dodecenoylcarnitine; C14, Tetradecanoylcarnitine; C14:1, Tetradecenoylcarnitine; C14:2, Tetradecadienoylcarnitine; C14OH, 3-Hydroxy-tetradecanoylcarnitine; C16, Hexadecanoylcarnitine; C16:1, Hexadecenoylcarnitine; C16OH, 3-Hydroxy-hexadecanoylcarnitine; C16:1OH, 3-Hydroxy-hexadecenoylcarnitine; C18, Octadecanoylcarnitine; C18:1, Octadecenoylcarnitine; C18:2, Octadecadienoylcarnitine; C18:OH, 3-Hydroxy-octadecanoylcarnitine; C18:1OH, 3-Hydroxy-octadecenoylcarnitine.

Targeted metabolomics was performed using MSMS. A total of 43 metabolites were analyzed. Radial graphics show the 11 amino acids, succinylacetone, and 31 acylcarnitines analyzed in our study (**Figures 1A, B**). Hierarchical clustering analysis was performed to identify changes in metabolite abundance among the study groups (**Figure 1C**) and showed that the most marked changes in average relative concentration occurred in 25 of the 43 studied metabolites. It is noteworthy to mention that concentrations of some metabolites changed as disease severity increased. Similar to amino acids associated to COVID-19 outcome, several short chain acylcarnitines, such as acetylcarnitine, and 3-hydroxybutyryl carnitine/malonylcarnitine were also elevated according to disease severity (**Figure 1B**).

#### PLS-DA

**Figure 2:**
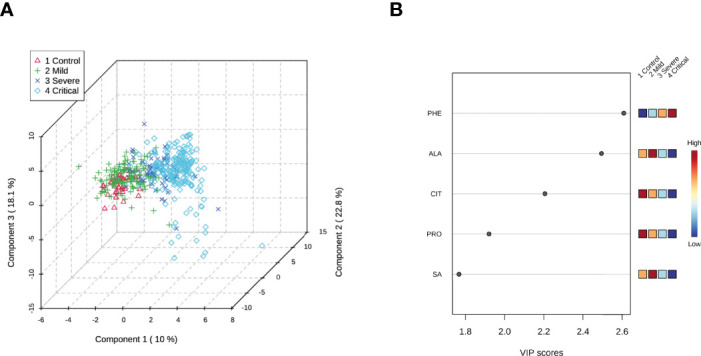
Three-dimensional score plot of selected components. **(A)** Partial least squares-discriminant analysis plot of differential metabolites from HS and patients with mild, severe, and critical COVID-19. The explained variances are shown in parentheses. **(B)** Metabolites with a variable importance in projection (VIP) score >1.5. The intensities of colors in boxes to the right (from blue to red) indicate the relative concentrations of the corresponding metabolite in each group under study. HS= healthy subjects. PHE, Phenylalanine; ALA, Alanine; CIT, Citrulline; PRO, Proline; SA, succinylacetone.

The presence of two clearly defined clusters was noted; one cluster comprised of healthy and ambulatory subjects and the other by hospitalized COVID-19 patients. Moreover, overlap within each cluster was observed (**Figure 2A**). A plot of VIP scores shows the contribution of each metabolite to the variability in each cluster (**Figure 2B**). A high VIP score indicates a greater contribution of the metabolite to separating the groups. We identified five metabolites with a VIP score >1.5 that were considered responsible for the observed separation. Phenylalanine had the highest VIP score (2.6), followed by alanine (2.5), citrulline (2.2), proline (1.9), and succinylacetone (1.7).

A paired PLS-DA was performed to refine the differences among the study groups (HS vs. mild; HS vs. severe; HS vs. critical; mild vs. severe; mild vs. critical, and severe vs. critical). As disease progressed toward the critical stage, the metabolomic differences became more apparent (**Figure 1S Supplementary Material**).

**Figure 3:**
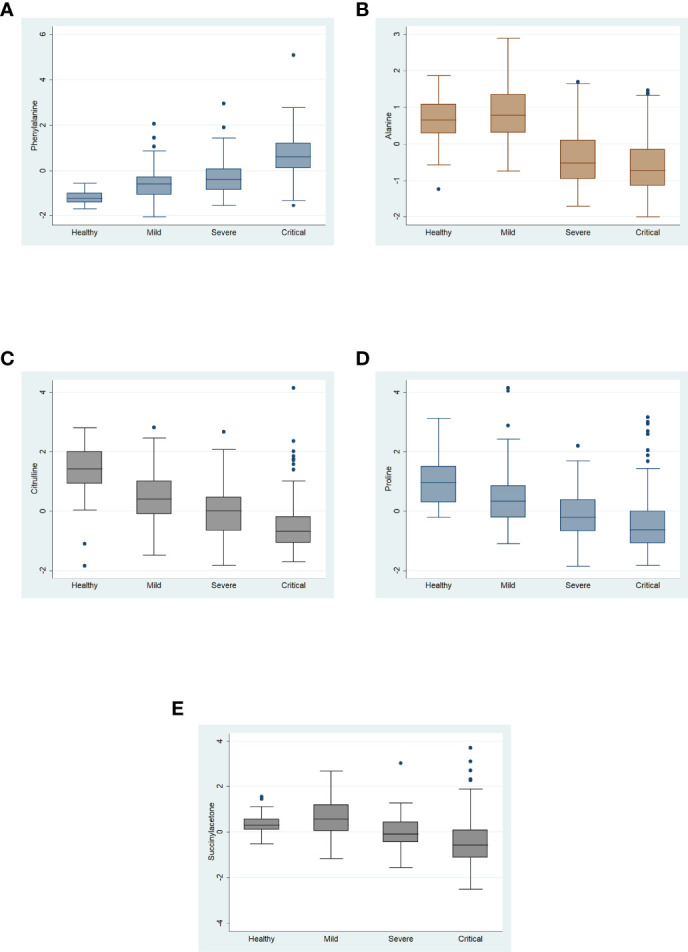
Normalized serum concentrations of the five metabolites with variable importance in projection of >1.5 from healthy subjects and patients with mild, severe, and critical COVID-19. Data are expressed as median with interquartile range.

**Figure 4:**
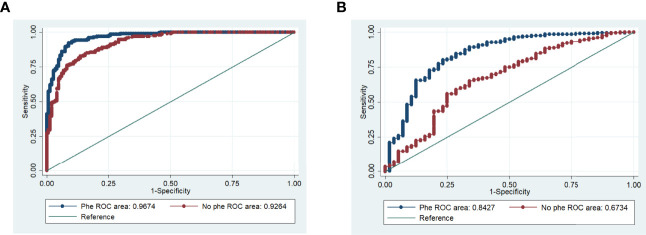
Receiver operating characteristic (ROC) curves for phenylalanine. **(A)** Phenylalanine in mild vs. critical model, adjusted by covariates. **(B)** Phenylalanine in severe vs. critical model, adjusted by covariates (all p ≤ 0.003).

**Figure 3** shows the normalized concentrations of the primary metabolites. Phenylalanine showed the lowest value in HS and the highest in patients with critical outcomes (**Figure 4A**), whereas citrulline and proline showed a tendency to decrease (**Figures 4C, D**). Alanine and succinylacetone levels were the highest in patients with mild disease, whereas a tendency toward decreased levels was observed in severe and critical COVID-19 patients (**Figures 4B–E**).

### Odds Ratios (ORs) of COVID-19 Outcomes Relative to Various Metabolites

In the unadjusted model the metabolites that showed a significant association with the severe COVID-19 group were phenylalanine, alanine, proline, and succinylacetone. The same metabolites plus citrulline were also found associated with the critical COVID-19 group. When comparing severe versus critical groups, a significant association was only observed for phenylalanine and citrulline. Regarding the adjusted model by age, sex, T2DM, hypertension, and BMI, we found statistically significant associations for alanine and succinylacetone with the severe COVID-19 group (OR=0.22; 95% CI 0.12-0.36 and OR=0.45; 95% CI 0.29-0.68, respectively). Moreover, for critical COVID-19 group, phenylalanine, alanine, citrulline, proline, and succinylacetone were associated. When comparing the severe versus critical COVID-19 groups significant associations for phenylalanine and citrulline were observed (OR=5.3; 95% CI=3.16-9.2 and OR=0.52; 95% IC=0.36-0.74, respectively) (**Table 2**).

**Table 2:** Unadjusted and adjusted logistic regression models for the association of metabolite profile with COVID-19 disease severity.

Metabolite	Severe			Critical			Severe vs. Critical		
	OR	IC 95%	P value	OR	IC 95%	P value	OR	IC 95%	P value
Unadjusted model									
Phenylalanine	2.23	1.39-3.58	0.001*	10.15	6.49-15.88	0.001*	4.7	2.92-7.66	<0.001*
Alanine	0.18	0.11-0.28	<0.001*	0.11	0.07-0.16	<0.001*	0.63	0.44-0.91	0.016
Citrulline	0.59	0.41-.84	0.004	0.23	0.16-0.32	<0.001*	0.48	0.34-0.68	<0.001*
Proline	0.56	0.39-0.80	0.001*	0.31	0.22-0.41	<0.001*	0.64	0.47-0.88	0.007
Succinylacetone	0.48	0.34-0.68	<0.001*	0.28	0.21-0.37	<0.001*	0.66	0.49-0.87	0.004
Adjusted model**									
Phenylalanine	1.62	0.95-2.78	0.07	8.4	4.89-14.45	<0.001*	5.3	3.16-9.2	<0.001*
Alanine	0.22	0.12-0.36	<0.001*	0.12	0.07-0.20	<0.001*	0.56	0.37-0.84	0.005
Citrulline	0.64	0.43-0.95	0.05	0.29	0.19-0.42	<0.001*	0.52	0.36-0.74	<0.001*
Proline	0.65	0.44-0.94	0.03	0.39	0.27-0.56	<0.001*	0.67	0.48-0.93	0.019
Succinylacetone	0.45	0.29-0.68	<0.001*	0.26	0.17-0.39	<0.001*	0.68	0.50-0.93	0.016

*Bonferroni test P=0.003. **Adjusted by age, sex, T2DM, hypertension, and nutritional status.

#### ROC Curve Analysis of the Relationships Between Metabolites and COVID-19 Outcomes

ROC curve analyses were performed to test each metabolite in the previously described logistic regression models. Among all metabolites, the AUC for the association of phenylalanine with critical COVID-19 group was 0.96 (95% CI=0.95-0.98) (**Figure 4A**). Additionally, ROC curve analysis comparing the relationship of phenylalanine with severe versus critical groups exhibited an AUC of 0.84 (95% CI=0.77-0.90) (**Figure 4B**).

## Discussion

Viral infections can provoke an imbalance in metabolic pathways such as those associated with energy and protein and lipids catabolism (20). During SARS-CoV-2 infection, changes in the metabolome could be related to disease outcome (17). In the present study, we explored the metabolome of COVID-19 patients to find differences associated with disease severity and to identify metabolites or metabolomic profiles that could serve as prognostic markers. Our metabolomic results showed a clear impact on amino acid metabolism, with significant increases in phenylalanine serum concentrations as disease severity progressed. In contrast, levels of alanine, citrulline, proline, decreased as the disease worsened.

Phenylalanine had the highest VIP score (2.6), showing an increase according to disease severity. This increase in phenylalanine concentration was previously reported in a longitudinal study (27) and in other studies of COVID-19 patients (20, 28–32) [20,27-31]. Moreover, in our logistic regression model adjusted by age, sex, T2DM, hypertension, and nutritional status, the significant elevation in levels of this amino acid was conserved (**Table 2**). These findings are supported by the ROC curve analyses, which showed an AUC of 0.96 with phenylalanine, compared with an AUC of 0.92 without phenylalanine. These results support the potential usefulness of phenylalanine as a biomarker of COVID-19 severity.

**Figure 5:**
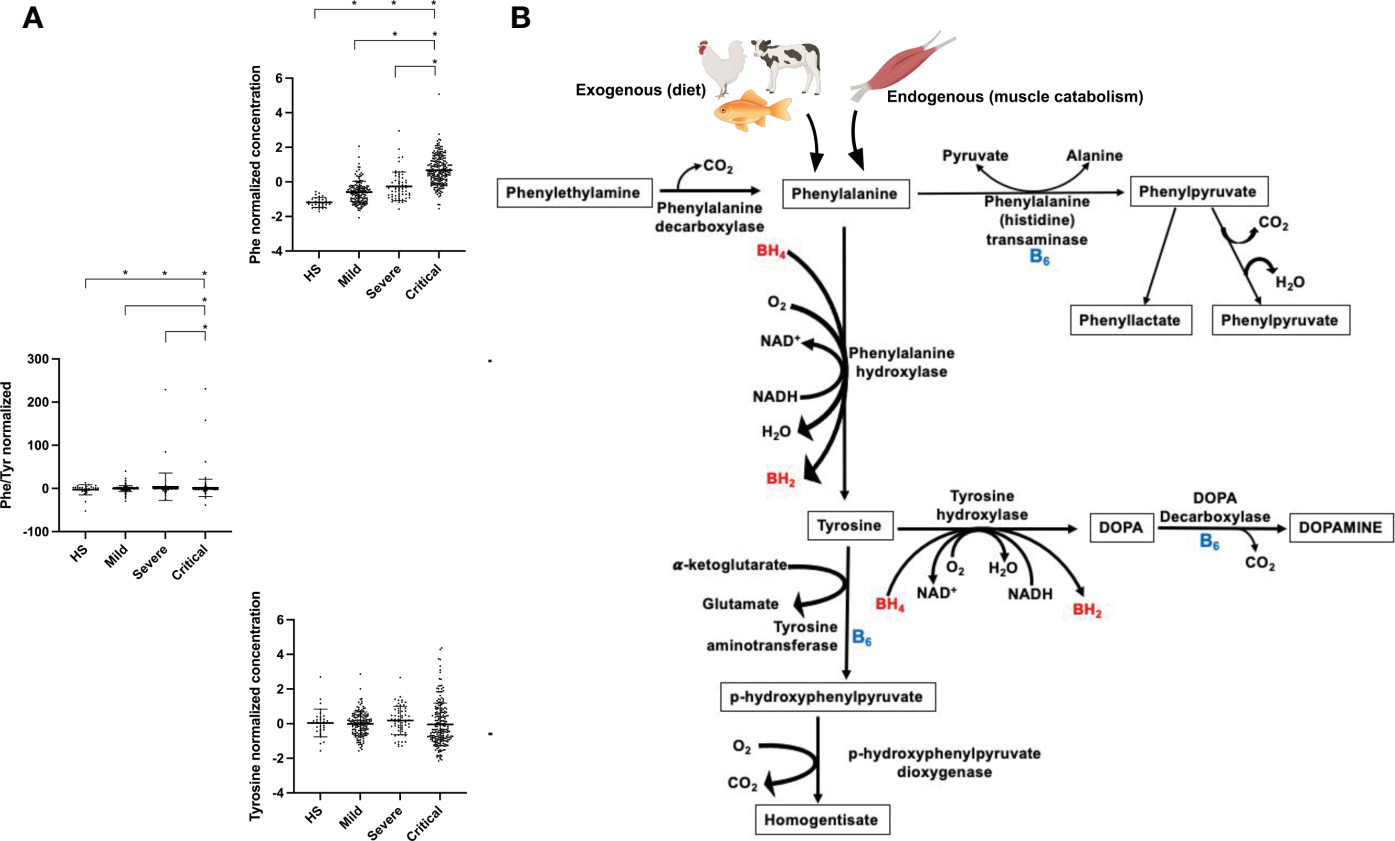
Phenylalanine **(A)** and tyrosine **(B)** metabolic pathways and observed metabolic changes in COVID-19 patients. AS, Aspartate synthetase; ASL, Argininosuccinate lyase; ARG, Arginase; NO, Nitric oxide; BH4, Tetrahydrobiopterin; BH2, Dihydrobiopterin; HS, Healthy subjects; SA, succinylacetone; TCA, tricarboxylic acid cycle.

Phenylalanine is an essential ketogenic and glucogenic amino acid and a precursor of tyrosine and dopamine-related neurotransmitters (33). In the present study, tyrosine showed a slight tendency to accumulate in severe and critical patients. The primary phenylalanine and tyrosine catabolic pathway is hydroxylation *via* the catalytic action of phenylalanine and tyrosine hydroxylases (34) (**Figure 5**). Both types of enzymes use molecular oxygen and tetrahydrobiopterin (BH4) as a cofactor (34, 35) [32,33]. In addition, nucleosides such as NADH are required for regeneration of the reduced form of BH4 (34) [32]. Thus, a lack of some of these factors could cause a deficiency in catalytic activity, thereby explaining the observed accumulation of both amino acids. Transamination is another mechanism by which phenylalanine and tyrosine can be catabolized *via* the action of phenylalanine and tyrosine transaminases, forming phenylpyruvate from phenylalanine and p-hydroxyphenylpyruvate from tyrosine (33). These enzymatic reactions use vitamin B6 (pyridoxine) as a cofactor (36). A deficiency of this vitamin has been observed in COVID-19 patients (37). Therefore, these findings also strengthen our hypothesis that phenylalanine and tyrosine accumulate due to impaired hydroxylation and transamination caused by a possible deficiency of cofactors such as BH4, NADH, oxygen, and vitamin B6. Increased levels of phenylalanine have also been found in patients with other viral infections, such as HIV, and these increases have been associated with immune activation, microvascular endothelial damage, and an increased risk of cardiovascular events (27, 38, 39). It is important to highlight that the presence of high blood concentration of phenylalanine, specially those higher than 360 μmol/L are neurotoxic and require a nutritional therapeutic intervention (34).

In the present study succinylacetone had a VIP score > 1.5. Until our knowledge, this organic acid has never been related to COVID-19 patients. However, its concentration did not show a tendency to increase or decrease according to the disease severity, and the dispersion of the data did not allow us to make any hypothesis or propose it as a potential biomarker of COVID-19 severity.

**Figure 6:**
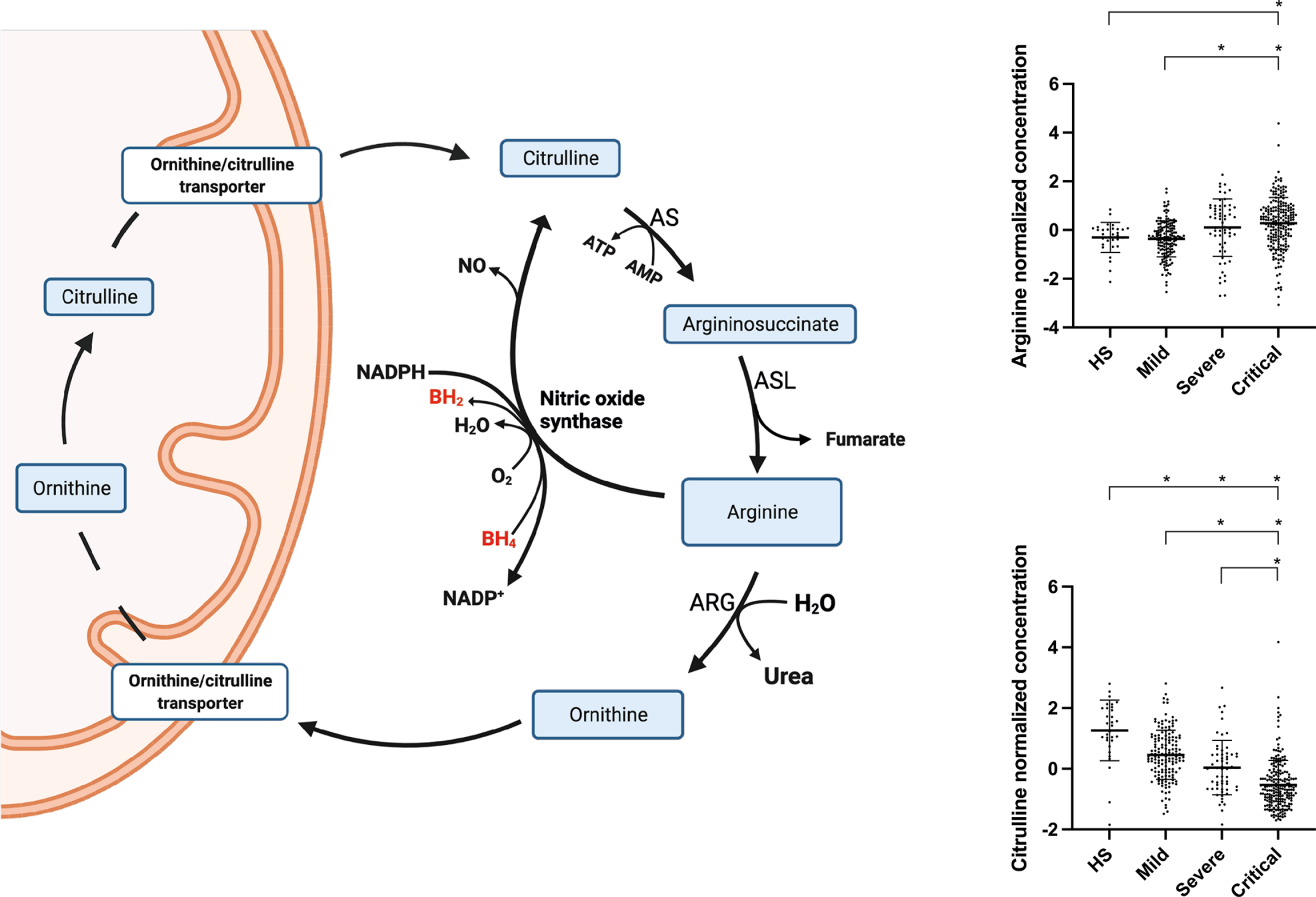
Arginine and citrulline metabolic pathways and observed changes in COVID-19 patients. BH4, Tetrahydrobiopterin; BH2, Dihydrobiopterin; B6, Pyridoxine; HS, Healthy subjects; PHE, Phenylalanine; TYR, Tyrosine.

Citrulline levels showed a significant decrease as COVID-19 worsened, and this difference was conserved in the adjusted model, accompanied by a tendency toward increased arginine levels (**Figure 6**). This increase in arginine levels in COVID-19 patients has also been reported by others, however the mechanism remains unclear (40). Arginine is metabolized to citrulline *via* nitric oxide synthase (NOS), an enzyme also dependent on BH4, oxygen, and NADPH (41). Thus, the observed accumulation of arginine and decrease in citrulline levels could also be explained by a deficiency of these cofactors. NOS activity plays an essential role in endothelial homeostasis, and its impairment can provoke cardiovascular events such as arterial hypertension (42). Arterial hypertension reportedly contributes to COVID-19 disease severity (27). Furthermore, hypertensive episodes have also been observed in severely ill COVID-19 patients (43, 44). These events could be triggered by an imbalance in the citrulline–nitric oxide cycle.

Our results show a significant decrease in alanine serum levels with worsening COVID-19. In this context, alanine gains importance as a glucogenic component of the Cahill cycle (also known as the glucose-alanine cycle) to supply energy demand (45). Alterations in alanine levels have a marked impact on muscle integrity; for example, decreased levels of alanine reduce the biosynthesis of carnosine, a critical dipeptide that prevents the muscle mass loss associated with sarcopenia (46). Furthermore, COVID-19 patients commonly exhibit high energy demand, skeletal muscle catabolism, and sarcopenia, which can increase their risk of death (47–49). Therefore, maintaining alanine homeostasis is crucial to decreasing the risk of severe disease (47, 48). Toward that end, interventions such as dietary alanine supplementation could reverse these pernicious effects of SARS-CoV-2 (50) [48].

We found an increase of the acetylcarnitine and 3-hydroxybutyryl/malonyl carnitine according to disease progression (**Figure 2**). Even though this finding was not significant; it deserves to be revised. Increased acylcarnitines in COVID-19 patients have been proposed as activators of proinflammatory pathways (51), and their imbalance has been related to ATP depletion (30). Our results support the fact that COVID-19 patients present an over utilization of lipid beta-oxidation pathway to supply the high energetic demand (52). Thus, this could also suggest an important dysregulation of these metabolites especially the short chain acylcarnitines, which are fundamental for maintaining the optimal energetic status.

Taken together, our results not only confirm the metabolomic findings of other studies (17, 20, 27, 53), they also highlight the importance of mechanisms such as the catalytic activity of phenylalanine and tyrosine hydroxylases, transaminases, and NOS enzymes, which are dependent on BH4, oxygen, NADH, NADPH, and vitamin B6, and the potential importance of deficiencies of these cofactors. These data could highlight the importance of nutritional interventions, such as vitamin B6 or BH4 supplementation as a strategy for improving the outcome of COVID-19 patients, as the energy demand increases with disease progression and metabolites related to exacerbation of catabolic pathway disruptions have been exhaustively reported in COVID-19 patients (52, 54). We propose the inclusion of phenylalanine as a plausible marker of COVID-19 severity, as diverse analytical methodologies such as MSMS are available for its efficient quantification on a large scale.

The major comorbidities in our study population were hypertension and T2DM, resulting in worse COVID-19 outcomes. The typical clinical features observed among COVID-19 patients in this study, such as fever, cough, dyspnea, chest pain, headache, odynophagia, rhinorrhea, myalgia, diarrhea, vomiting, abdominal pain, and anosmia, were similar to hallmarks reported in other studies (5, 55). Other prominent clinical manifestations described in COVID-19 patients are low oxygen saturation (SpO2) and hypoxemia (56–58). In this sense, the tendency toward low SpO2 in our study could be related to illness severity. Critical subjects showed a SpO2 level of 83% (IQR 71-88); such a low SpO2 level in combination with anemia could induce hypoxemia affecting the respiratory system, which is the main characteristic of severe outcomes in COVID-19 (56).

The present study has some limitations, mainly its cross-sectional design, which precluded the possibility of monitoring metabolites in conjunction with disease progression. Thus, further prospective studies are needed to characterize changes in the COVID-19 metabolome as disease severity progresses. Another limitation is that some of the measured metabolites are molecular species with the same nominal mass but different exact masses, and this isobaric characteristic avoided to distinguish them with the MS/MS methodology used. Finally, the nutrition of the patients at moment of the sample collection was not possible to determine, considering that all the samples were taken at the triage facilities or in the first hours of hospital admission.

In conclusion, the metabolomic fingerprint of COVID-19 related to disease progression is characterized by dysregulation of amino acids and short chain acylcarnitines metabolic pathways, especially those of aromatic amino acids. Which would be related to a deficiency in cofactors such as BH4, vitamin B6 and nucleosides (NADH and NADPH), which are essential for hydroxylation, transamination and monooxygenation enzymatic reactions. Our data suggest that a metabolic dysregulation could induce states of hypoxemia, loss of endothelial function, and other clinical damage characteristic of COVID-19. The deregulation of amino acids such as phenylalanine, alanine, citrulline, and proline observed in our study was clearly associated with critical outcomes in COVID-19 patients. Therefore, phenylalanine could be considered as a promising biomarker of COVID-19 severity.

## Data Availability Statement

The datasets presented in this study can be found in online repositories. The names of the repository/repositories and accession number(s) can be found below: [INSERT REPOSITORY AND ACCESSION].

## Ethics Statement 

The studies involving human participants were reviewed and approved by the ethic committee of Instituto Nacional de Rehabilitacion Luis Guillermo Ibarra Ibarra (INR-LGII: 17/20). The patients/participants provided their written informed consent to participate in this study.

## Author Contributions

AL-R, MV-A and LM-G: study conception and design. LM-G, II-G, CF-L, CM-A, GJ-G, PV-C, MC-R, and CS-A: literature search.TT,PV-V, JR-H, AR-Z, GR-V, FM-R, MM, DZ-A, EB-G, GL-C, and NG-F: included patients. LM-G, GV, FS-M, RP-S, IC-Z, MV-V, and GM-N: acquisition of data. CF-L, SO-P, BH-L, JO-T, MJ-A, MR-V, and JM: performed experiments. LM-G, II-G, HM-M, TT, CP, MV-A, GM-N, and AL-R: data analysis and interpretation. SC-N, CF-L, and CM-A: figures. LM-G, II-G, CF-L, MV-A, and AL-R: drafting the article. TT, GV, CP, MV-A, IC-Z, and AL-R: critical revision of the article and final approval of the version to be published. All authors contributed to the article and approved the submitted version.

## Funding

This study was funded by the Consejo Nacional de Ciencia y Tecnología; CONACYT 312513 SARS-COV 2, and National Institute of Pediatrics (Recursos Fiscales 2018-2020, Programa E022 Investigación y Desarrollo Tecnológico en Salud, Ciudad de México, México, Protocol number 2021/008-C).

## Conflict of Interest

The authors declare that the research was conducted in the absence of any commercial or financial relationships that could be construed as a potential conflict of interest.

## Publisher’s Note

All claims expressed in this article are solely those of the authors and do not necessarily represent those of their affiliated organizations, or those of the publisher, the editors and the reviewers. Any product that may be evaluated in this article, or claim that may be made by its manufacturer, is not guaranteed or endorsed by the publisher.

## Supplementary Material

Supplementary Figure 1 Two-dimensional score plots of selected principal components, according to disease severity.
