# Supplementary material for: Metabolic Reprogramming in SARS-CoV-2 Infection Impacts the Outcome of COVID-19 Patients
Source: Front Immunol. 2022 Jul 11;13:936106. doi: 10.3389/fimmu.2022.936106 (PMC9634751; doi:10.3389/fimmu.2022.936106)
Supplement: Supplementary Figure 1 — Two-dimensional score plots of selected principal components, according to disease severity. [file Image_1.pdf]

Supplementary Material

1 Supplementary Figures

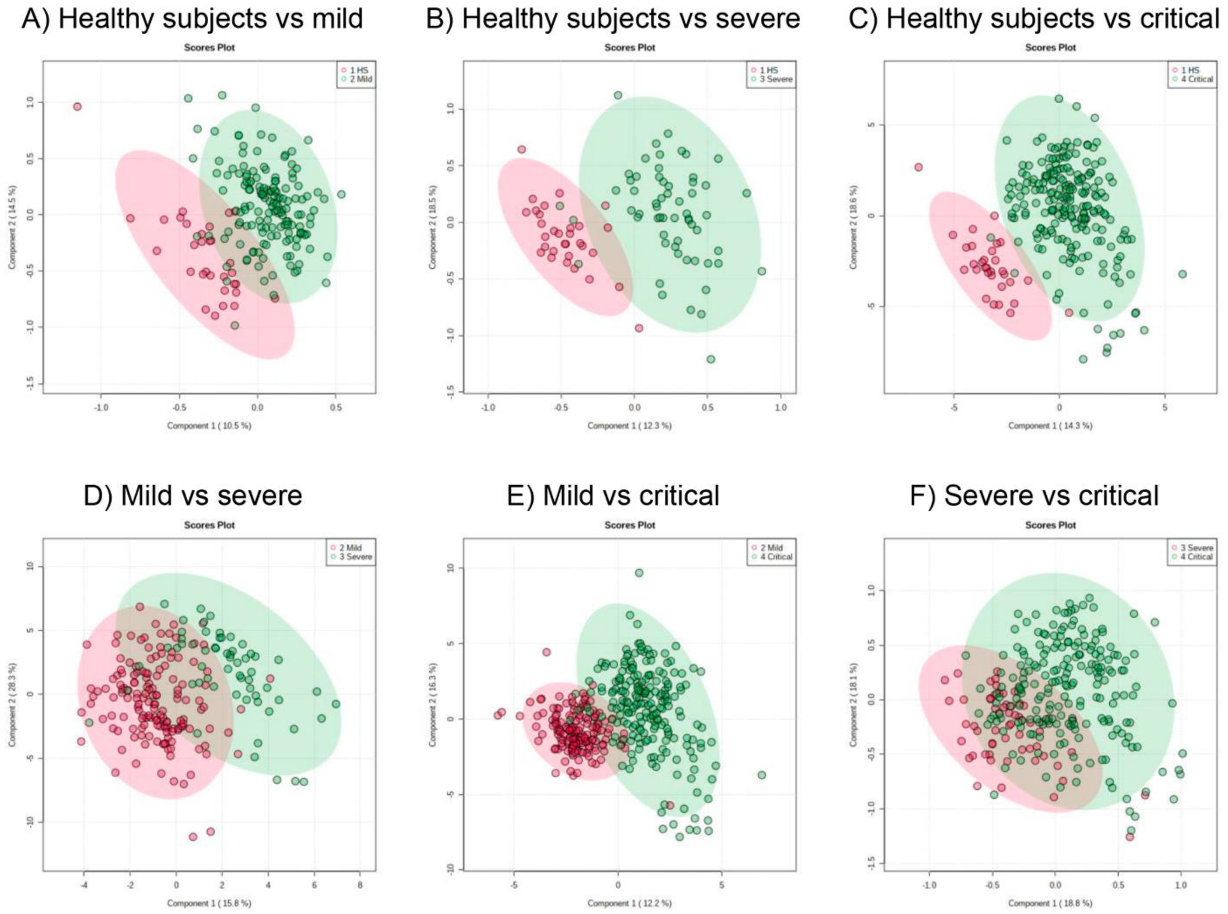

Supplementary Figure 1. Two-dimensional score plots of selected principal components, according to disease severity. The explained variances are shown in brackets
